# Ultra-dense lutetium oxide ceramic scintillators for positron emission tomography

**DOI:** 10.1088/1361-6560/ae456f

**Published:** 2026-02-23

**Authors:** Akram Hamato, Daehee Lee, Ryosuke Ota, Hamidreza Hemmati, Matthias Muller, Yimin Wang, Lakshmi Soundara Pandian, Suyoung Kim, Taiga Yamaya, Simon R Cherry, Jarek Glodo, Sun Il Kwon

**Affiliations:** 1 Department of Biomedical Engineering, University of California, Davis, One Shields Avenue, Davis, CA 95616, United States of America; 2 Central Research Laboratory, Hamamatsu Photonics K.K, 5000, Hirakuchi, Hamana-ku, Hamamatsu 434-8601, Japan; 3 Radiation Monitoring Devices, Inc., Watertown, MA 02472, United States of America; 4 Institute for Quantum Medical Science, National Institutes for Quantum Science and Technology, 4-9-1 Anagawa, Inage-ku, Chiba 263-8555, Japan

**Keywords:** ceramic scintillator, Lu_2_O_3_:Yb, (Lu, Y)_2_O_3_:La, PET, time-of-flight PET

## Abstract

*Objective.* Lutetium oxide (Lu_2_O_3_), with its high density (9.4 g cm^−3^), presents a compelling scintillation host for detecting 511 keV annihilation photons in positron emission tomography (PET). Despite its favorable density, the practical deployment of Lu_2_O_3_-based scintillators for PET has faced limitations due to difficulties in crystal growth and inappropriate decay time. Recent progress in ceramic processing has facilitated the development of transparent Lu_2_O_3_ ceramics, while targeted doping strategies have significantly improved their luminescence performance. This study evaluates the performance of Lu_2_O_3_:Yb and a newly developed ceramic scintillator of (Lu,Y)_2_O_3_:La, a modified Lu_2_O_3_-based compound incorporating yttrium (Y) and doped with lanthanum (La). *Approach.* Various ceramic disks were fabricated and cut into 3 × 3 × 5 mm^3^ samples. The performance of both Lu_2_O_3_:Yb and (Lu,Y)_2_O_3_:La ceramic samples in terms of decay time, energy resolution, and coincidence timing resolution (CTR) was assessed. Decay time measurements were conducted using waveform data collected from samples mounted on an H10580 photomultiplier tube (PMT) and irradiated with 511 keV photons from a ^22^Na source. Energy and CTRs were evaluated using both PMT and silicon photomultiplier setups, arranged in coincidence with a reference lutetium–yttrium oxyorthosilicate (LYSO) detector of the same size. *Main results.* All three (Lu,Y)_2_O_3_:La ceramic scintillator samples exhibited a triple exponential decay profile and were dominated by a slow component ranging from 1379.3 to 1515.6 ns. The best energy resolution of 15.4% at 511 keV and the best CTR of 237.9 ps full width at half maximum (FWHM) were observed for the same sample. In contrast, a fast decay time of 1.6 ns was observed for the Lu_2_O_3_:Yb samples, which exhibited CTR values ranging from 237.9 ps to 261.4 ps FWHM, while the photopeak at 511 keV was difficult to distinguish. These CTR values were estimated between two identical ceramic samples, derived from coincidence measurements of each ceramic sample against the LYSO reference detector. The (Lu,Y)_2_O_3_:La samples achieved CTR values comparable to those of the Lu_2_O_3_:Yb samples, as their much higher light yield offsets the disadvantage associated with their slower decay time. *Significance.* These results highlight the promising potential of the (Lu,Y)_2_O_3_:La ceramic scintillators for PET applications, especially for time-of-flight PET.

## 1. Introduction

Scintillators are widely used in medical imaging, particularly in positron emission tomography (PET) scanners, due to their ability to convert high-energy photons into visible light. Materials such as lutetium oxyorthosilicate (Lu_2_SiO_5_, commonly referred to as LSO), and lutetium–yttrium oxyorthosilicate (LYSO)(Lu_2(1−*x*)_Y_2*x*_SiO_5_ where *x* denotes the fraction of yttrium replacing lutetium, commonly referred to as LYSO) have been popular for their high density, effective stopping power, and fast decay times, making them well-suited for the precise detection required in PET systems (Melcher 2000, Valais *et al* 2007). Compared to LSO, LYSO offers improved crystal growth stability, reduced production cost, and slightly enhanced mechanical durability, while preserving comparable scintillation performance (Pepin *et al* 2004). The growth of these crystals typically relies on the Czochralski method, which involves high-temperature melting, the use of crucible materials, and precise control of dopant concentrations. These requirements make the fabrication process technically demanding and economically intensive (Melcher 2000, Pepin *et al* 2004, Cai *et al* 2018). Therefore, the relatively high cost (Qin *et al* 2005) and complex manufacturing processes of LYSO and LSO have driven interest toward alternative scintillators, particularly ceramic-based materials.

Early ceramic scintillators were confined to thin-plate formats due to their intrinsic opacity, limiting their practical applications. Advances in shaping and sintering technologies have since enabled the fabrication of transparent ceramics with enhanced chemical stability and flexible rare-earth ion doping capabilities (Liu *et al* 2024). Furthermore, ceramic scintillators can achieve greater thickness than monocrystalline forms through sintering, a method that minimizes crack formation during fabrication and enables more uniform dopant distribution. The maximum attainable dimensions of ceramic scintillators are limited by the sintering furnace capacity (Yanagida *et al* 2005).

Lutetium oxide (Lu_2_O_3_) has the potential to become a highly promising scintillation host material for PET applications due to its exceptionally high density (∼9.4 g cm^−3^), which is significantly greater than that of LYSO (7.1 g cm^−3^), and its high effective atomic number (68) (Miller *et al* 2004). These distinct properties of Lu_2_O_3_ enable superior stopping power for 511 keV photons. Compared to conventional scintillators like LSO or LYSO, this allows thinner Lu_2_O_3_ ceramics to achieve equivalent stopping power, thereby reducing material volume and cost. Thinner detectors typically improve light collection and therefore energy resolution and timing resolution, as well as reducing off-center resolution losses via the depth of interaction effect (Yang *et al* 2008). However, the high melting point of Lu_2_O_3_ (2490 °C) (SpringerMaterials 2024) hinders traditional single-crystal growth, making bulk scintillator fabrication costly and impractical for large-scale production (Wang *et al* 2024). If a scintillation material has a cubic structure, this limitation can be overcome through ceramic processing techniques that consolidate refractory powders into optically transparent forms (Wisniewski *et al* 2008, Glodo *et al* 2017, Pandian *et al* 2023). Compared to conventional single crystals, ceramic scintillators offer greater scalability and simpler fabrication processes, making them a more cost-effective solution for large-volume or complex geometries. These advantages make ceramic scintillators increasingly appealing for modern PET scanner designs, where affordability and performance must be carefully balanced (Glodo *et al* 2025).

The choice of dopant is critically important, as undoped Lu_2_O_3_ lacks the necessary light emission centers to efficiently convert absorbed 511 keV photon energy into detectable scintillation photons. Therefore, the incorporation of specific rare-earth ions as dopants has been extensively studied to develop ceramic scintillators with efficient light output for various applications (Miller *et al* 2004, Taira *et al* 2014, Yanagida *et al* 2014, 2024, Hu *et al* 2022, Viers *et al* 2024, Glodo *et al* 2025). Among these, Lu_2_O_3_:Eu has been developed for high-resolution x-ray imaging, both as powder-based scintillator screens (Miller *et al* 2004) and as transparent thin films (Babayevskaya *et al* 2010, Cha *et al* 2012, De Jesus Morales Ramírez *et al* 2013, Seferis *et al* 2014), although its relatively slow scintillation decay, typically on the order of hundreds of microseconds, remains a limitation for fast imaging applications. Ytterbium-doped Lu_2_O_3_ (Lu_2_O_3_:Yb) has received considerable attention for its exceptionally fast decay time, typically in the range of 1.1–1.7 ns, which positions this ceramic scintillator as a leading candidate for future time-of-flight (TOF) application in high-energy physics experiments (Taira *et al* 2014, Hu *et al* 2022, Zhang and Zhu 2025). This scintillator can also be considered as a candidate for next-generation TOF PET (Glodo *et al* 2024), where precise timing resolution is crucial for enhancing signal-to-noise ratio (SNR) in the reconstructed PET image. However, the reported light yield (LY) of Lu_2_O_3_:Yb, which ranges from 500 to 2000 photons/MeV, is significantly lower than that of LYSO (32 000 photons/MeV) (Glodo *et al* 2024, Qin *et al* 2025), thereby limiting the precise measurement of gamma-ray energy in PET. Although Lu_2_O_3_:Yb has been the subject of only preliminary investigations into its optical and scintillation properties, to the best of our knowledge no systematic study has been reported on its potential application in PET.

While Lu_2_O_3_:Yb offers an ultrafast decay time yet suffers from low LY, lanthanum-doped Lu_2_O_3_ (Lu_2_O_3_:La) has demonstrated higher LY with an acceptably long decay time, characteristics that make it a strong candidate for PET applications (Glodo *et al* 2025), where high sensitivity and timing resolution are crucial. A good energy resolution (less than 10%) at 511 keV photons and the timing resolution of approximately 290–330 ps against lanthanum bromide (LaBr_3_) have been reported for 2 mm-thick Lu_2_O_3_:La (Glodo *et al* 2024, 2025).

In our recent studies, we focused on improving the brightness of Lu_2_O_3_:Yb (Kwon *et al* 2023) and refining the manufacturing methods of Lu_2_O_3_:La (Hemmati *et al* 2024, Glodo *et al* 2025), with the aim of making both scintillators more viable options for PET scanners. For this study, we fabricated several new (Lu,Y)_2_O_3_-based scintillators, which are oxide materials combining lutetium (Lu) and yttrium (Y), doped with lanthanum (La). Lanthanum functions as a scintillation center by localizing an exciton, while the inclusion of yttrium enhances the solubility of La in the lattice, which is otherwise limited in pure Lu_2_O_3_. Although combining Lu and Y slightly reduces stopping power compared to Lu_2_O_3_, it makes the material more cost-effective and improves production yield. In this study, fundamental characteristics such as decay time, energy resolution, and coincidence timing resolution (CTR) were evaluated to determine the suitability of both Lu_2_O_3_:Yb and newly developed (Lu,Y)_2_O_3_:La samples fabricated using ceramic processing. These parameters are crucial for assessing the performance of Lu_2_O_3_ as a scintillator material for advanced PET scanners.

## 2. Materials and methods

**Figure 1.**
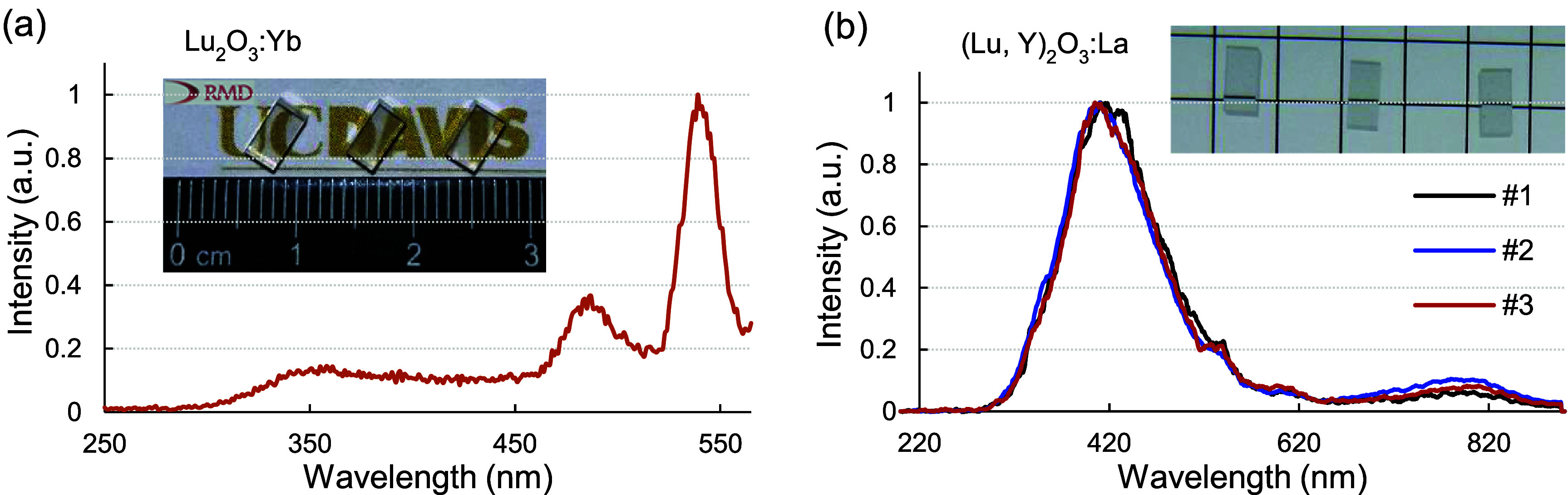
Emission spectra of (a) a representative Lu_2_O_3_:Yb sample and (b) the (Lu,Y)_2_O_3_:La samples. Photographs of the three samples of Lu_2_O_3_:Yb and (Lu,Y)_2_O_3_:La are also shown in panels (a) and (b), respectively.

Ceramic scintillators of Lu_2_O_3_:Yb, with the composition (Lu_0.99_Yb_0.01_)_2_O_3_, and the newly developed composition (Lu,Y)_2_O_3_:La, with the formula (Lu_0.77_Y_0.17_La_0.06_)_2_O_3_, were fabricated in disk form by Radiation Monitoring Devices Inc. (Watertown, MA, USA) using established ceramic processing techniques. One representative disk was selected and cut into small pieces of 3 × 3 × 5 mm^3^, and the surfaces of each piece were mechanically polished. For both types of scintillators, a variety of samples were prepared to evaluate their performance stability. Lu_2_O_3_:Yb samples have an effective atomic number of 68 and a density of 9.4 g cm^−3^, whereas (Lu,Y)_2_O_3_:La samples exhibit an effective atomic number of 67 and a density of 8.6 g cm^−3^. The emission spectra under x-ray excitation of a representative Lu_2_O_3_:Yb and the three (Lu,Y)_2_O_3_:La samples used in this study are presented in figure 1. The emission peaks of the Lu_2_O_3_:Yb sample are around 350–370 nm, which are consistent with the previously reported values (Taira *et al* 2014, Yanagida *et al* 2014, Zhang and Zhu 2025), and at 480 nm and 540 nm. The peaks observed at 480 nm and 540 nm are caused by impurities, possibly Tb, with decay times around a millisecond (Taira *et al* 2014). The emission peaks of the (Lu,Y)_2_O_3_:La samples appear around 420 nm and 800 nm. Photographs of the samples are also included in figure 1. The top and lateral surfaces of all samples were wrapped in three layers of polytetrafluoroethylene tape to maximize light collection efficiency and optimize detector performance.

Two types of detectors were assembled using different photo-sensors: a fast photomultiplier tube (PMT, H10580, Hamamatsu Photonics K.K., Japan; 25 mm diameter, super bialkali; rise time: 1 ns) and a silicon photomultiplier (SiPM, AFBR-S4N44P014M, Broadcom, USA; effective area 3.7 × 3.8 mm^2^). Gain of both photo-sensors, quantum efficiency (QE) of the PMT and photon detection efficiency (PDE) of the SiPM are given in table 1 (Hamamatsu 2014, Broadcom 2024). Each sample was coupled to the center of the active area of its corresponding photosensor using a very thin layer of silicon grease, applied to the unwrapped surface to ensure optimal optical contact.

**Table 1.** Typical gain and quantum efficiency (QE) of a Hamamatsu H10580 PMT, and typical gain and photon detection efficiency (PDE) of a Broadcom AFBR‐S4E0001 SiPM.

	Hamamatsu H10580 PMT	Broadcom AFBR‐S4E0001 SiPM
Gain	6 × 10^5^ @ high voltage of 1200 V	7.3 × 10^6^ @ bias voltage of 44.5 V
QE/PDE	27% @ 420 nm	65% @ 420 nm and bias voltage of 48 V

### 2.1. Decay time measurement

**Figure 2.**
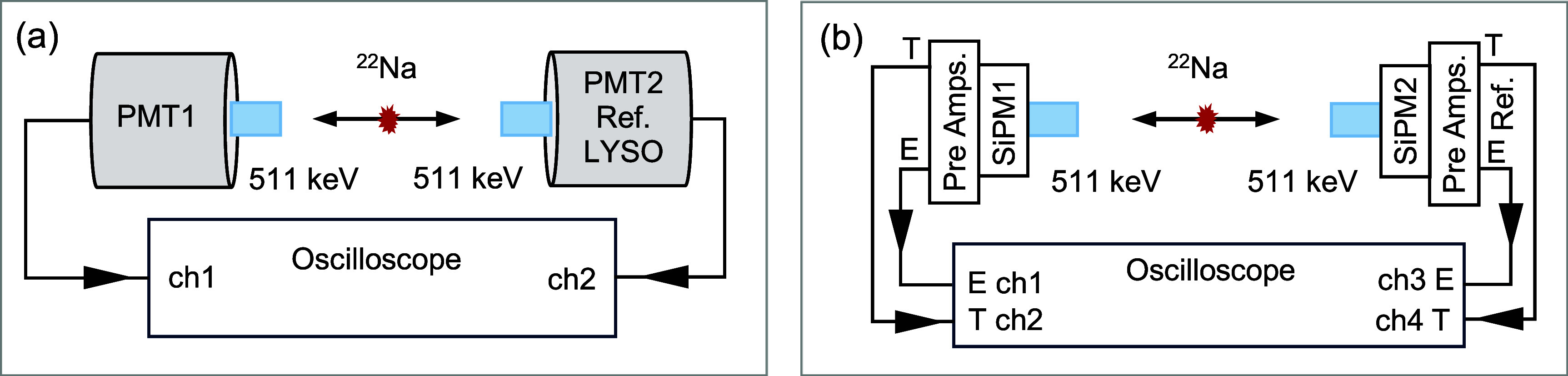
Experimental setups for (a) energy and decay time measurements and (b) energy and timing performance evaluation.

Decay curves were measured with the ceramic samples coupled to the fast PMT biased at −1200 V and irradiated with a ^22^Na point source. The measurements were performed using a coincidence setup with a reference detector consisting of a LYSO crystal (3 × 3 × 5 mm^3^) coupled to an identical H10580 PMT. The scintillation pulses from detected coincidence events arising from 511 keV annihilation photon interactions in both detectors were captured using an 8 GHz bandwidth oscilloscope (MSO 70804C, Tektronix, USA) with a 40 ps bin size (figure 2(a)). The average decay times of each sample were determined by fitting the measured waveforms with tri-exponential functions.

### 2.2. Energy and timing performance

Energy resolution at 511 keV was evaluated for both photo-sensor types, with each scintillator sample coupled either to the PMT (figure 2(a)) or to the SiPM (figure 2(b)). Each SiPM was connected to a custom pole-zero cancellation preamplifier board (AFBR-S4E0001, Broadcom, USA) to generate both energy and timing signals, which were digitized using the oscilloscope (figure 2(b)). The reference detector consisted of a LYSO crystal (3 × 3 × 5 mm^3^) coupled to an identical SiPM. The bias voltage of the SiPM on the reference side was set to 37 V, while a higher bias of 48 V was applied to the SiPM coupled to the samples in order to achieve optimal timing performance.

To derive the energy spectrum, the baseline of each waveform was first subtracted, followed by integration of the entire area under the full corrected waveform, ensuring no part of the signal was excluded. The energy resolution of each sample was evaluated for both photo-sensor types by fitting the 511 keV photopeak using a Gaussian function and calculating the ratio of the full width at half maximum (FWHM) to the photopeak centroid.

CTR for each sample against the LYSO reference sample, hereafter referred to as CTR, determined by fitting a dual-Gaussian function to data acquired with the SiPM detector setup (figure 2(b)). Events were selected within an energy window of ±0.5 × FWHM around the 511 keV photopeak for the reference detector and the (Lu,Y)_2_O_3_:La detectors, whereas custom-defined energy windows, independent of energy spectrum fitting, were applied to the Lu_2_O_3_:Yb detectors to mitigate the influence of low LY. The effect of bias voltage on CTR was evaluated for a Lu_2_O_3_:Yb sample by changing the SiPM bias voltage of the test detector from 37 V to 48 V. Single timing resolution (STR) of LYSO reference detector was 111.8 ps.

## 3. Results

### 3.1. Decay time

**Figure 3.**
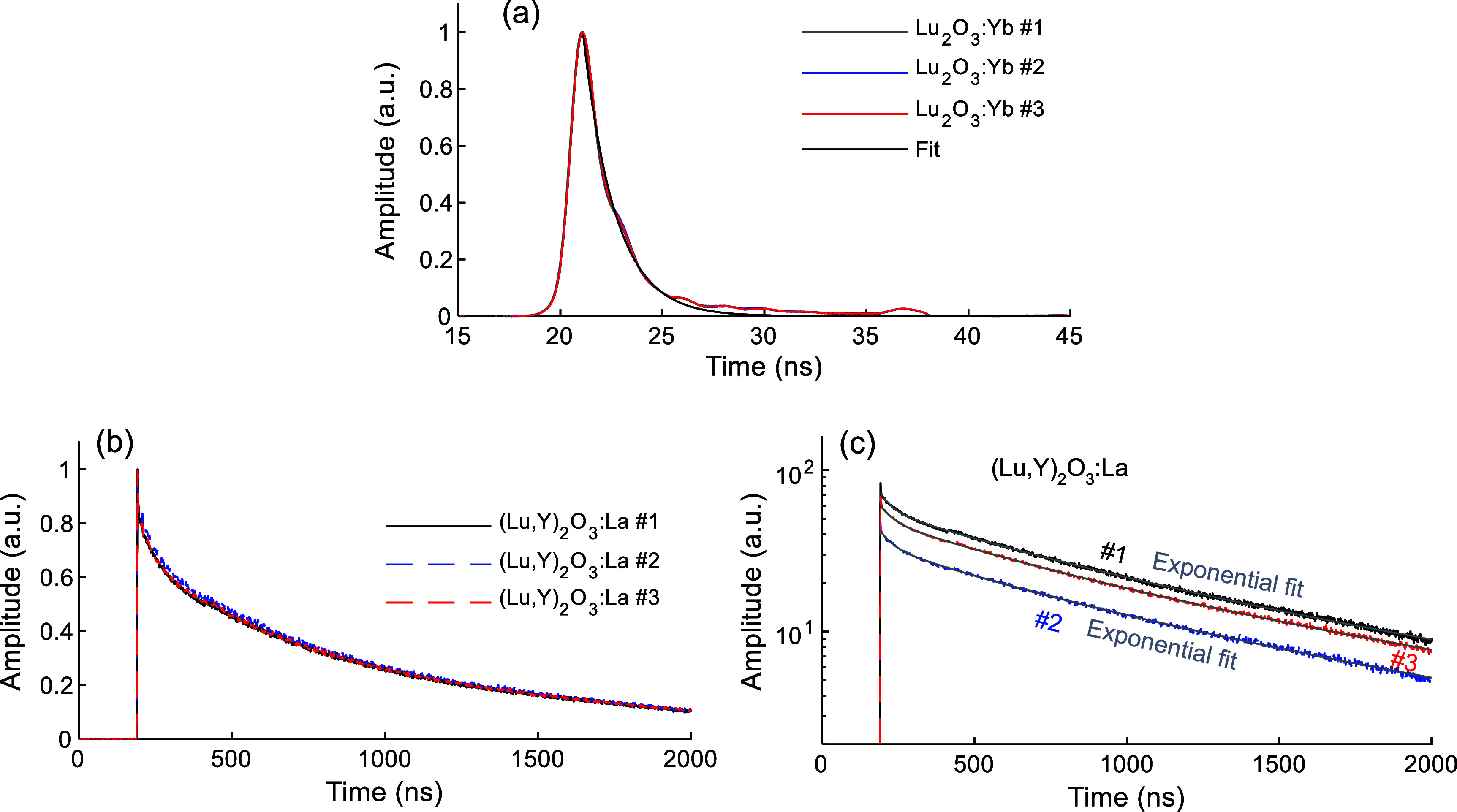
Average waveforms of (a) Lu_2_O_3_:Yb and (b) (Lu,Y)_2_O_3_:La samples in linear scale, and (c) (Lu,Y)_2_O_3_:La samples in logarithmic scale, compared to fitted decay curves. Measurements were done using the PMT setup illustrated in figure 2(a).

Average waveforms of 511 keV photons were measured using the PMT (figure 2(a)) for all samples. Each average waveform was generated by averaging 1000 individual events to improve the statistical quality for decay time estimation. Figure 3(a) compares the results for three Lu_2_O_3_:Yb samples, each curve fitted with a single-component exponential function, yielding a main decay component of 1.6 ns across all three. The average waveforms for three (Lu,Y)_2_O_3_:La samples are presented in figure 3(b), with fitted curves for all samples shown in figure 3(c). Each waveform was fitted using a sum of three exponential functions representing fast, medium, and slow decay components. The decay times and their relative intensity fractions for all samples are summarized in table 2.

**Table 2.** Decay times for Lu_2_O_3_:Yb samples, and fast (*τ*_f_), medium (*τ*_m_) and slow (*τ*_s_) decay components along with their corresponding fractions for (Lu,Y)_2_O_3_:La samples.

Scintillator	Sample ID	#1	#2	#3
Lu_2_O_3_:Yb	*τ* (ns)	1.6	1.6	1.6

(Lu,Y)_2_O_3_:La	*τ*_f_ (ns)	40.1	44.0	41.6
	*τ*_m_ (ns)	498.4	445.3	491.1
	*τ*_s_ (ns)	1489.7	1379.3	1515.6
	Fast component fraction (%)	1.2%	1.1%	1.0%
	Medium component fraction (%)	25.5%	21.3%	25.3%
	Slow component fraction (%)	73.3%	77.6%	73.7%

While Lu_2_O_3_:Yb samples demonstrated ultrafast decay times, slower decay behaviors were observed in the (Lu,Y)_2_O_3_:La samples. The fast decay components for all (Lu,Y)_2_O_3_:La samples ranged from 40.1 to 44.0 ns with light fractions around approximately 1% for all three samples. The medium decay components fell between 445.3 and 498.4 ns, with fractions ranging from 21.3% to 25.5%. In contrast, the slow decay components were in the range of 1379.3–1515.6 ns, accounting for the highest contribution (73.3% to 77.6%). These decay characteristics are comparable with previously reported values for Lu_2_O_3_:La scintillators (Glodo *et al* 2025).

### 3.2. Energy performance

**Figure 4.**
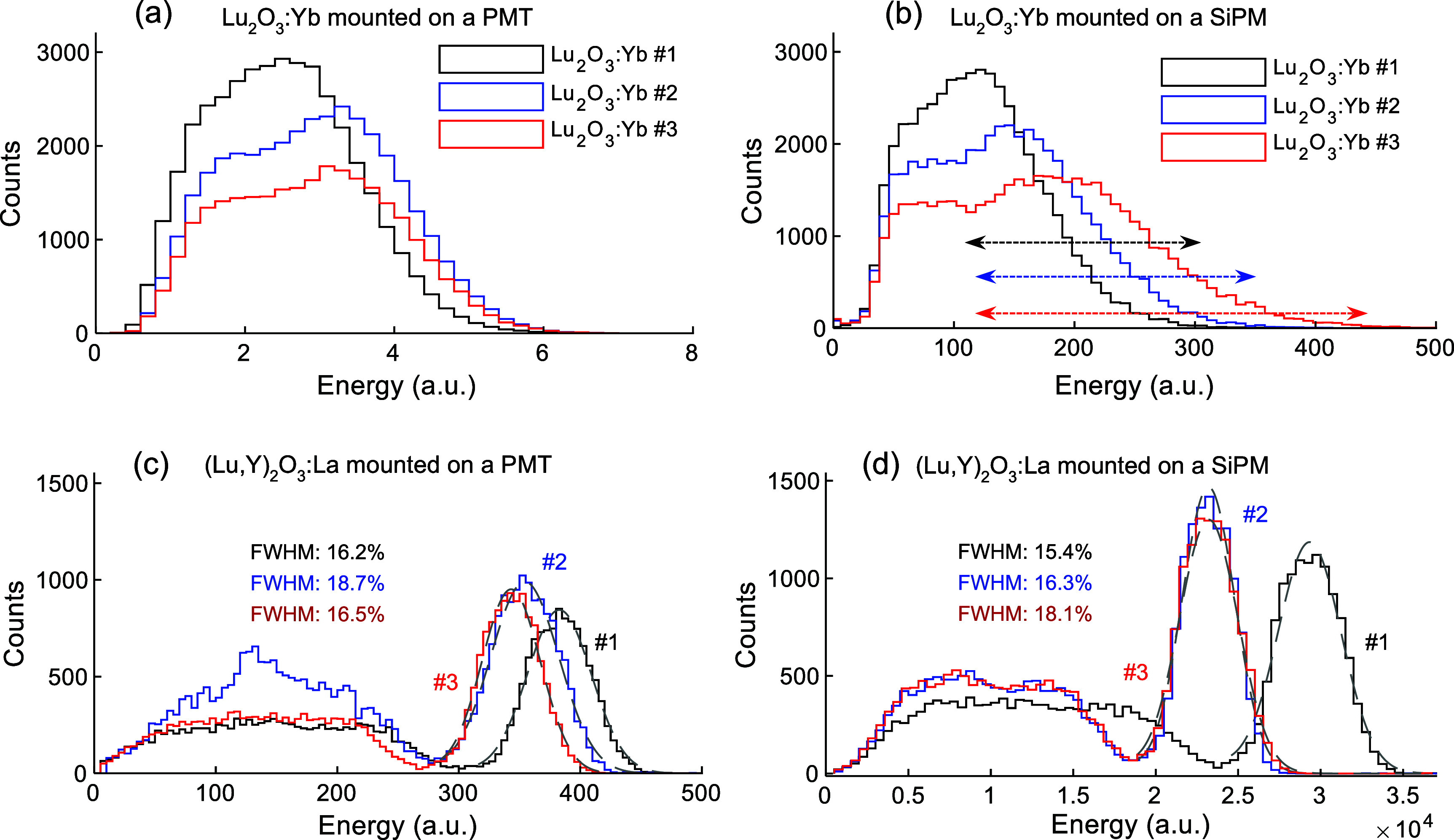
Energy spectra of three Lu_2_O_3_:Yb samples measured with (a) a PMT and (b) a SiPM. Energy spectra of (Lu,Y)_2_O_3_:La samples measured with (c) a PMT and (d) a SiPM, including fitted 511 keV photopeaks for all three samples. Detector setups are shown in figure 1, with bias voltages of 1200 V and 48 V applied to the ceramic side for the PMT and SiPM measurements, respectively. The horizontal arrows in (b) show the applied energy windows used for CTR evaluation for Lu_2_O_3_:Yb samples.

The energy spectra for Lu_2_O_3_:Yb and (Lu,Y)_2_O_3_:La samples measured with both photo-sensor types are shown in figures 4(a)–(d). The (Lu,Y)_2_O_3_:La samples exhibit higher LY and correspondingly much better energy resolution than the Lu_2_O_3_:Yb samples.

The spectra obtained for the Lu_2_O_3_:Yb samples with both photo-sensors (figures 4(a) and (b)) differ noticeably, which is attributed to the mismatch between the Lu_2_O_3_:Yb emission peak (350–370 nm) and the peak PDE or QE of both photo-sensors (420 nm). In contrast, the emission peak of (Lu,Y)_2_O_3_:La samples matched with the peak PDE or QE of the photo-sensors. The higher LY of (Lu,Y)_2_O_3_:La samples compared to that of Lu_2_O_3_:Yb arises from the more efficient scintillation centers provided by La compared to Yb. The energy resolutions of the Lu_2_O_3_:Yb samples are poor due to their low LY.

Table 3 presents the energy resolutions and photopeak values at 511 keV for the (Lu,Y)_2_O_3_:La samples mounted on the PMT and SiPM, respectively. No results were reported for the Lu_2_O_3_:Yb samples, as a photopeak was not clearly distinguishable (figures 4(a) and (b)). For comparison, table 3 also includes the energy resolution and photopeak value for the reference detector composed of a LYSO crystal coupled to the PMT.

**Table 3.** Energy resolutions and photopeak values at 511 keV for (Lu,Y)_2_O_3_:La samples and a LYSO crystal measured with the PMT at a bias voltage of 1200 V, and for (Lu,Y)_2_O_3_:La samples measured with the SiPM at a bias voltage of 48 V.

	Sample ID	#1	#2	#3	LYSO
PMT	Energy resolution (%)	16.2 ± 0.3	18.7 ± 0.3	16.5 ± 0.3	13.5 ± 0.2
	Photopeak value (a.u.)	382.8 ± 0.5	354.3 ± 0.4	343.2 ± 0.4	541.6 ± 0.4

SiPM	Energy resolution (%)	15.4 ± 0.4	16.3 ± 0.4	18.1 ± 0.8	—
	Photopeak value (a.u.)	29 339 ± 48	23 202 ± 34	23 214 ± 73	—

For the (Lu,Y)_2_O_3_:La samples mounted on either the PMT or SiPM, energy resolutions at 511 keV ranged from 15.4% to 18.7%, with the best performance observed for sample ID #1 coupled to the SiPM. The energy resolution for sample ID #3 degraded slightly when coupled to the SiPM compared with the PMT while only minor improvements were observed for samples ID #1 and #2. These differences could be attributed to slight variations in the coupling conditions when the samples were measured with the PMT and the SiPM.

**Figure 5.**
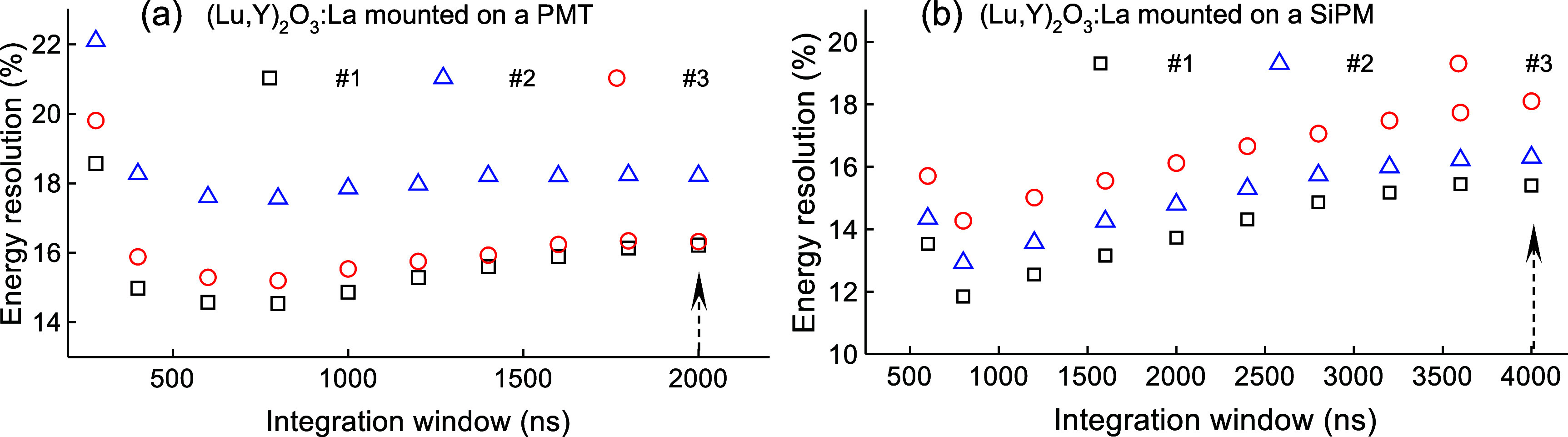
Energy resolutions at 511 keV as a function of integration window for (Lu,Y)_2_O_3_:La samples measured with the (a) PMT at a fixed high voltage of 1200 V and (b) SiPM at a fixed bias voltage of 48 V. The vertical arrow inside the plot indicates the energy resolution obtained with the full integration window for the spectra in figures 4(c) and (d), with the base of the arrow corresponding to the exact value associated with the entire integration window.

The energy resolutions of the (Lu,Y)_2_O_3_:La samples mounted on both PMT and SiPM were further evaluated using varying integration windows to explore potential improvements. Figure 5 presents the energy resolution of the samples as a function of integration window. For all samples, narrowing the integration window led to improved energy resolution, with the effect being particularly larger for those mounted on the SiPM. For sample ID #1, the resolution improved from 16.2% to 14.5% on the PMT when the integration range was reduced from 0–2000 ns to 0–800 ns, and from 15.4% to 11.8% on the SiPM when the integration range was reduced from 0–4000 ns to 0–800 ns.

### 3.3. Timing performance

**Figure 6.**
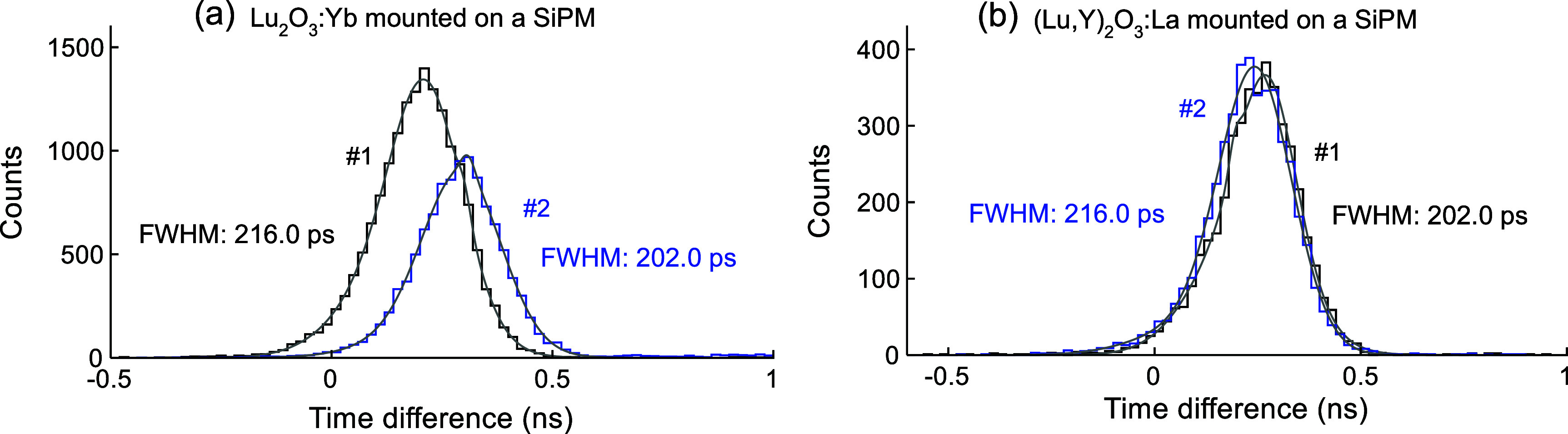
Timing spectra measured between each sample and the LYSO reference detector (figure 1(b)) for (a) Lu_2_O_3_:Yb and (b) (Lu,Y)_2_O_3_:La samples, both measured with the SiPM.

Timing spectra for four samples, Lu_2_O_3_:Yb (ID #1 and #2) and (Lu,Y)_2_O_3_:La (ID #1 and #2), mounted on the SiPM are shown in figure 6. The SiPM bias voltage was set to 48 V, and energy windows of ±0.5 × FWHM around the photopeak were applied to both the reference detector and the detector incorporating the (Lu,Y)_2_O_3_:La samples. For the Lu_2_O_3_:Yb samples, the energy window was applied around the 511 keV photopeak as shown in figure 4(b) by the horizontal arrows. The shape of the timing spectra for all samples of both Lu_2_O_3_:Yb and (Lu,Y)_2_O_3_:La is asymmetric, as shown in figure 6, due to the use of a reference detector composed of LYSO scintillator rather than the identical ceramic material used in the detector under investigation. The reduced number of counts observed for sample #2 in figure 6(a) arises from its shorter measurement duration relative to sample #1. The best CTR of 202.0 ps FWHM was achieved for both the Lu₂O₃:Yb (ID #2) and (Lu,Y)₂O₃:La (ID #1) samples.

**Figure 7.**
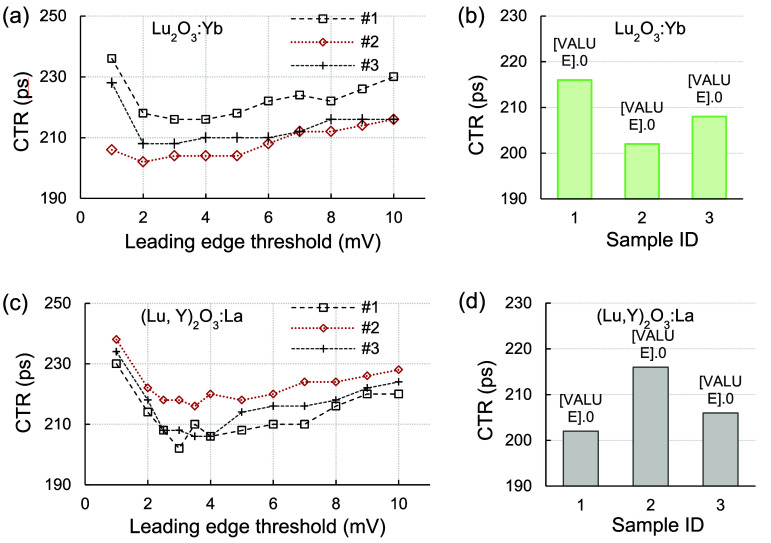
(a) Coincidence timing resolutions (CTRs), measured against a LYSO reference detector, as a function of leading-edge threshold for Lu_2_O_3_:Yb samples; (b) comparison of the best measured CTRs across all samples; (c) CTRs, measured against a LYSO reference detector, as a function of leading-edge threshold for (Lu,Y)_2_O_3_:La samples; (d) comparison of the best measured CTRs for the samples. Each sample was measured with the SiPM at a fixed bias voltage of 48 V.

Changes in CTR as a function of the leading-edge threshold are presented in figures 7(a) and (c) for the Lu_2_O_3_:Yb and (Lu,Y)_2_O_3_:La samples, respectively. The leading-edge threshold for the reference detector was set to 5 mV. The best CTRs for two Lu_2_O_3_:Yb samples (IDs #1 and #3) were achieved with a leading-edge threshold of 3 mV, whereas the best CTR for sample #2 was obtained at 2 mV. The average CTR for all three samples was 208.7 ± 7.0 ps. The best CTRs for the three (Lu,Y)_2_O_3_:La samples were observed at leading-edge thresholds ranging from 2 mV to 4 mV. The best value of 202.0 ps was achieved with sample #1 at the leading-edge threshold of 2 mV, while three samples exhibited an average CTR of 208.0 ± 7.2 ps. The best measured CTRs are compared in figures 7(b) and (d) for both scintillator types.

**Figure 8.**
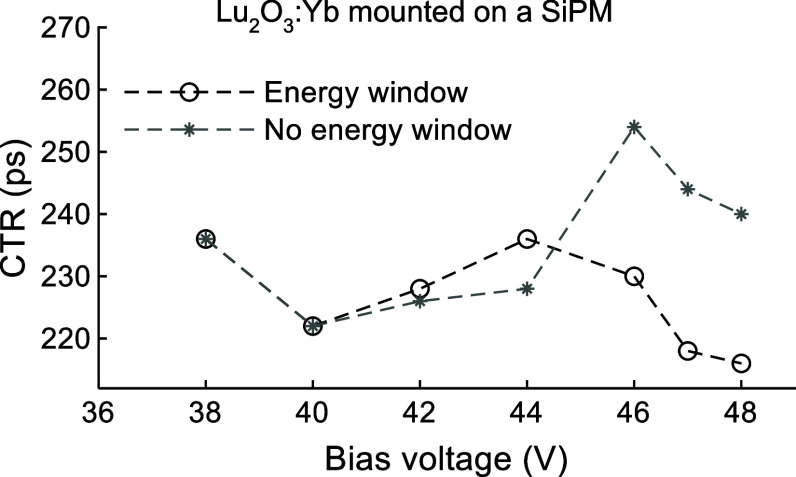
Coincidence timing resolutions (CTRs), measured against a LYSO reference detector, versus bias voltage and energy window for a Lu_2_O_3_:Yb sample. Measurements were performed with the sample mounted on the SiPM, as shown in figure 2(b). The horizontal arrows in figure 4(b) show the applied energy windows for the Lu_2_O_3_:Yb sample.

For the Lu_2_O_3_:Yb sample (ID #1), the effects of the energy window and bias voltage on the CTR are presented in figure 8. The best CTR was achieved at the highest bias voltage of 48 V when the energy window was applied. The CTR improved from 236.0 ps to 216.0 ps as the bias voltage increased from 38 V to 48 V. Additionally, applying an energy window around the 511 keV photopeak resulted in an improved timing resolution (figure 8), as evidenced by a reduction in CTR from 240.0 ps to 216.0 ps at a bias voltage of 48 V.

The CTR between two identical ceramic samples (CTR_iden_) can be calculated from the CTR of each ceramic sample against the LYSO reference detector, ${\mathrm{CT}}{{\mathrm{R}}_{{\mathrm{sample}} - {\mathrm{LYSO}}}}$, which is equal to the reported CTR in this paper, and the STR of the LYSO reference detector, ${\mathrm{ST}}{{\mathrm{R}}_{{\mathrm{LYSO}}}}$ (STR_LYSO_ = 111.8 ps), by $\sqrt 2 \times \sqrt {{\mathrm{CTR}}_{{\mathrm{sample}} - {\mathrm{LYSO}}}^2 - {\mathrm{STR}}_{{\mathrm{LYSO}}}^2} $. The estimated CTR_iden_ values for Lu_2_O_3_:Yb ceramic samples (#1, #2, and #3) were 261.4 ps, 237.9 ps, and 248.0 ps, respectively, while those for the (Lu,Y)_2_O_3_:La samples (#1, #2, and #3) were 237.9 ps, 261.4 ps, and 244.7 ps, respectively. These timing performances of both Lu_2_O_3_:Yb and (Lu,Y)_2_O_3_:La samples are comparable, with slight variations across individual samples.

## 4. Discussion

This study focuses on evaluating the performance of newly developed (Lu,Y)_2_O_3_:La ceramic scintillators in terms of decay time, energy resolution, and CTR, in direct comparison with Lu_2_O_3_:Yb, for their potential application in PET scanners. The decay time of Lu_2_O_3_:Yb was measured to be 1.6 ns (figure 2(a)), which is exceptionally fast and makes it a compelling candidate for high-rate applications such as high-energy physics and calorimetry. This ultrafast decay behavior is attributed to charge-transfer luminescence associated with Yb^3+^ ions (Hu *et al* 2022). In this process, electron capture by the Yb center from the oxygen valence band leads to parity-allowed transitions with high oscillator strength, resulting in rapid and efficient scintillation. The measured decay time of 1.6 ns aligns well with previously reported values in the literature, which typically range from 1.1 to 1.7 ns (Hu *et al* 2022, Qin *et al* 2025).

Decay time measurements of both Lu_2_O_3_:Yb and (Lu,Y)_2_O_3_:La ceramic scintillators were performed using a PMT, which provided sufficient time resolution to capture the fast scintillation components. To improve measurement fidelity, microchannel plate PMTs (MCP-PMTs) may be utilized due to their inherently faster single photon response (Ota *et al* 2021). This faster response enhances the SNR and enables more precise extraction of decay time parameters in fast-decay scintillators such as Lu_2_O_3_:Yb, as demonstrated by the ultrafast decay time of ∼1.1 ns measured with an MCP-PMT setup (Hu *et al* 2022), and in moderate light-output materials like (Lu,Y)_2_O_3_:La. Even with an MCP-PMT setup, intrinsic time resolution and signal shape of the MCP-PMT are convolved with scintillation kinetics. Therefore, the intrinsic decay time of Lu_2_O_3_:Yb can be sub-nanosecond after deconvolving the photosensor’s temporal properties.

To estimate the relative LY of (Lu,Y)_2_O_3_:La scintillators, photopeak values of the samples were compared to that of a LYSO crystal with identical dimensions (3 × 3 × 5 mm^3^), measured under the same conditions. Photopeak positions (table 2) for samples ID #1, and LYSO were 383, and 541, respectively, indicating that sample produced approximately 71% of LYSO’s light yield. Using similar estimates, samples ID #2 and #3 generated 65% and 63% of LYSO’s light yield, respectively. Assuming a LY of 30 000 ph MeV^−1^ for LYSO, the roughly estimated LYs are between 19 010 and 21 200 ph MeV^−1^ for three samples. The samples exhibit a LY similar to that of Lu_2_O_3_:La scintillators (approximately 20 000 ph MeV^−1^) (Glodo *et al* 2025). Applying the same estimation method to the Lu_2_O_3_:Yb samples, using approximate photopeak values of 2.5–3.7 a.u. Extracted from figure 4(a), yielded an estimated LY of up to 205 ph MeV^−1^, which is reasonably consistent with the reported value of 280 ph MeV^−1^ for a similar ceramic scintillator (Hu *et al* 2022). The difference arises from the rough nature of our estimation and from considering the same QE values for both Lu_2_O_3_:Yb and LYSO, despite the emission peak of Lu_2_O_3_:Yb (350–370 nm) being mismatched with the PMT response.

All CTR values against the reference LYSO detector were analyzed using a dual-Gaussian function, rather than a single Gaussian, to more accurately model each spectrum and extract the FWHM. This approach was necessary due to the inherent asymmetry observed in the CTR distributions (figure 6), which arose from the use of non-identical detectors in coincidence, causing asymmetric probability density function of scintillation emission (Kwon *et al* 2019, Tao *et al* 2022, Ota *et al* 2025).

CTR_iden_, coincidence timing resolution for two identical crystals, exhibits a direct dependence on the square root of the effective decay time and an inverse dependence on the squares root of the LY (Gundacker *et al* 2020). Accordingly, Lu_2_O_3_:Yb and (Lu,Y)_2_O_3_:La samples exhibited similar CTR_iden_, resulting from the very fast decay time of Lu_2_O_3_:Yb and the high light yield of (Lu,Y)_2_O_3_:La. The best CTR (202.0 ps), which corresponds to CTR_iden_ of 237.9 ps, was observed for Lu_2_O_3_:Yb sample #2 and (Lu,Y)_2_O_3_:La sample #1, measured at leading-edge thresholds of 2 mV and 3 mV, respectively (figure 7). The effective decay time ($1/{\tau _{{\mathrm{eff}}}} = \sum\nolimits_i {R_i}/{\tau _{di}}{ }$, where *R_i_* is the ratio of each fast, medium, and slow component) and the estimated LY for (Lu,Y)_2_O_3_:La sample #1 were 774.6 ns and 21 200 ph MeV^−1^, respectively. The relation of CTR_iden_ with effective decay time (*τ*_eff_) and LY, as $CT{R_{{\mathrm{iden}}}} \propto \sqrt {{\tau _{{\mathrm{eff}}}}/LY} $, for the sample #1 was ($\sqrt {774.6/21200} $) 0.2 while it was approximately ($\sqrt {1.6/190} $) 0.1 for Lu_2_O_3_:Yb sample #2. This apparent inconsistency is expected because the simplified scaling neglects several important factors such as differences in rise time, spectral mismatch between the scintillation emission and the SiPM PDE, scintillation self‐absorption, and photodetector noise. All these factors can influence the number and timing of the earliest detected photons and consequently CTR_iden_.

**Figure 9.**
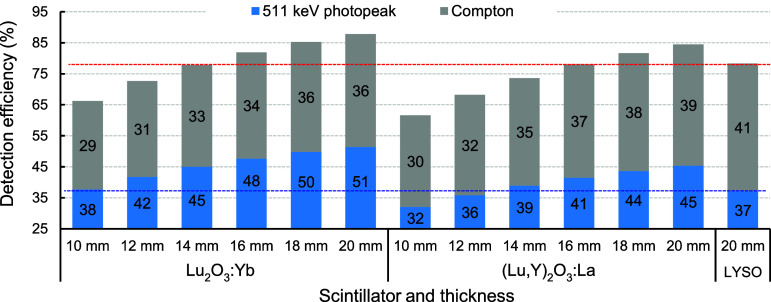
Simulated overall detection efficiency (in %) including both 511 keV photopeak and Compton interactions for Lu_2_O_3_:Yb and (Lu,Y)_2_O_3_:La scintillators with 3 × 3 mm^2^ cross-sections and thicknesses ranging from 10 to 20 mm, compared to a 3 × 3 × 20 mm^3^ LYSO crystal. Red and blue horizontal dashed lines indicate the overall and 511 keV photopeak efficiencies, respectively. All simulations were performed using the PHITS code under identical irradiation conditions.

To support the cost-reduction strategy of replacing LYSO with ultra-dense Lu_2_O_3_-based ceramic scintillators in PET systems, we conducted Monte Carlo simulations using the Particle and Heavy Ion Transport code System (PHITS) to calculate overall detection efficiency of 511 keV photons including both 511 keV photopeak and Compton interactions in a 3 × 3 × 20 mm^3^ LYSO crystal. Subsequently, we evaluated the required thicknesses of Lu_2_O_3_:Yb and (Lu,Y)_2_O_3_:La ceramics, using identical 3 × 3 mm^2^ cross-sectional dimensions, to achieve equivalent 511 keV photopeak and overall efficiencies in a single crystal. Simulations were performed under identical irradiation conditions for ceramic scintillators with thicknesses ranging from 10 mm to 20 mm, in 2 mm increments. The results in figure 9 indicate that Lu_2_O_3_:Yb and (Lu,Y)_2_O_3_:La ceramics with thicknesses of 10 mm and 13 mm, respectively, yield 511 keV photopeak efficiencies comparable to that of the 20 mm-thick LYSO crystal. These findings suggest that thinner ceramic scintillators can maintain sufficient sensitivity at the 511 keV photopeak, reinforcing their viability for PET applications at significantly reduced cost, further supported by the lower production expenses of ceramic processing. The overall detection efficiencies, defined as the combination of all 511 keV photopeak absorption and Compton events, were also compared in the figure. To achieve the same overall detection efficiency as the 20 mm LYSO, Lu_2_O_3_:Yb and (Lu,Y)_2_O_3_:La require thicknesses of 14 mm and 16 mm, respectively, while the Compton scattering fraction is lower in the ceramic scintillators. The simulation also demonstrated that photoelectric absorption fractions to the total interactions were 38%, 35% and 33% for Lu_2_O_3_:Yb, (Lu,Y)_2_O_3_:La and LYSO, respectively. Additionally, the ceramic scintillators exhibit lower Compton interaction fractions, which may reduce inter crystal scattering when implemented as arrays in PET scanners, thereby improving image quality.

Despite LYSO being the standard scintillator material for current PET systems, Lu_2_O_3_-based scintillators such as Lu_2_O_3_:Yb and (Lu,Y)_2_O_3_:La, with their higher density and comparable effective atomic number, enables the use of substantially thinner scintillation elements while maintaining comparable detection sensitivity. This reduction in crystal thickness markedly decreases the amount of expensive scintillator raw material required and lowers the associated cost of ceramic processing. The combined effect of reduced material consumption and more economical fabrication by ceramic processing offers significant potential for lowering the overall cost of PET instrumentation. Furthermore, the thinner crystal geometry can mitigate parallax error, thereby improving spatial resolution, particularly in PET systems without depth-of-interaction capability (Levin and Hoffman 1999, Razdevšek *et al* 2025). While both ceramic scintillators are promising for reducing cost and mitigating parallax error in PET scanners compared to LYSO, further improvements in light yield, decay time, and timing performance are still required to achieve competitive system‐level performance as PET detectors.

The performance of (Lu,Y)_2_O_3_:La demonstrates an interesting balance between promising detector metrics and intrinsic limitations associated with its relatively slow scintillation kinetics. Although the material exhibits an effective decay time of 700–800 ns and a slow component extending to approximately 1500 ns, it still achieves a CTR_iden_ of 237.9 ps. This notable CTR_iden_ is supported by the relatively high light yield of (Lu,Y)_2_O_3_:La which provides a sufficiently strong prompt signal despite the presence of slow decay components. However, the long effective decay time remains a potential drawback for high‐count-rate PET environments, where extended scintillation pulses may increase pulse pile‐up probability, elevate detector dead time, and reduce overall system throughput. Therefore, careful design of front-end electronics and system architecture may be required to enable PET scanners based on (Lu,Y)_2_O_3_:La to operate effectively in high-count-rate environments, with reference to previously reported methods (Amsel *et al* 1969, Wang *et al* 2012).

Overall, the findings demonstrate that both Lu_2_O_3_:Yb and (Lu,Y)_2_O_3_:La ceramics, still under development, offer complementary advantages for PET applications: the former excels in timing characteristics due to its ultrafast decay, while the latter delivers much higher light yield and better energy resolution. The ability to fine-tune detector performance through bias voltage and signal integration strategies underscores the versatility of ceramic scintillators in next-generation imaging systems. Continued efforts to optimize dopant concentrations are underway to further enhance scintillator properties and achieve performance on par with, and eventually exceeding, commercial systems based on LYSO. These developments mark a promising step toward scalable, high-performance alternatives for clinical PET imaging.

## 5. Conclusion

This study evaluated the performance of Lu_2_O_3_ ceramic scintillators doped with Yb and newly developed (Lu,Y)_2_O_3_ ceramic scintillators doped with La, using samples of 3 × 3 × 5 mm^3^. The Lu_2_O_3_:Yb samples exhibited a fast decay time of 1.6 ns, while the (Lu,Y)_2_O_3_:La samples showed tri-exponential decay behavior. All three samples were dominated by a slow component between 1379.3 and 1515.6 ns with a 73.3%–77.6% contribution. A clearly distinguishable 511 keV photopeak was observed in all (Lu,Y)_2_O_3_:La samples, with the best energy resolution of 15.4%. Optimizing the waveform integration window improved this energy resolution to 11.8%. The best CTR of 237.9 ps for two identical scintillators was achieved for the sample with the energy resolution of 15.4%. The 511 keV photopeak was difficult to resolve in the Lu_2_O_3_:Yb samples; however, good CTRs ranging from 237.9 to 261.4 ps were observed.

These findings demonstrate the potential of (Lu,Y)_2_O_3_:La ceramics for PET systems, particularly TOF PET applications, offering promising opportunities for performance optimization and significant cost reduction in large-scale applications such as total-body.

## Data Availability

The data cannot be made publicly available upon publication because they are not available in a format that is sufficiently accessible or reusable by other researchers. The data that support the findings of this study are available upon reasonable request from the authors.
